# Risk of colorectal cancer among immigrants to Ontario, Canada

**DOI:** 10.1186/s12876-017-0642-5

**Published:** 2017-07-06

**Authors:** Lawrence Paszat, Rinku Sutradhar, Ying Liu, Nancy N. Baxter, Jill Tinmouth, Linda Rabeneck

**Affiliations:** 1 University of Toronto, Institute for Clinical Evaluative Sciences, G106 2075 Bayview Avenue, Toronto, ON M4N3M5 Canada; 2 University of Toronto, St Michael’s Hospital, 30 Bond Street, Toronto, ON M5B1W8 Canada; 3 University of Toronto, Sunnybrook Health Sciences Centre, 2075 Bayview Avenue, Toronto, ON M4N3M5 Canada; 4 University of Toronto, Prevention and Cancer Control, Cancer Care Ontario, 620 University Avenue, Toronto, ON M4 Canada

**Keywords:** Colorectal cancer, Cancer registry, Immigrants, Exposure-control matched design, Cox proprotional hazards regression

## Abstract

**Background:**

The risk of colorectal cancer (CRC) varies around the world and between females and males. We aimed to compare the risk of CRC among immigrants to Ontario, Canada, to its general population.

**Methods:**

We used an exposure-control matched design. We identified persons in the Immigration, Refugees and Citizenship Canada Permanent Resident Database with first eligibility for the Ontario Health Insurance Plan between July 1, 1991 and June 30, 2008 at age 40 years or older, and matched five controls by year of birth and sex on the immigrant’s first eligibility date. We identified CRC from the Ontario Cancer Registry between the index date and December 31, 2014. All analyses were stratified by sex. We calculated crude and relative rates of CRC. We estimated risk of CRC over time by the Kaplan-Meier method and compared immigrants to controls in age and sex stratified strata using log-rank tests. We modeled the hazard of CRC using Cox proportional hazards regression, accounting for within-cluster correlation by a robust sandwich variance estimation approach, and assessed an interaction with time since eligibility.

**Results:**

Among females, 1877 cases of CRC were observed among 209,843 immigrants, and 16,517 cases among 1,049,215 controls; the crude relative rate among female immigrants was 0.623. Among males, 1956 cases of CRC were observed among 191,792 immigrants and 18,329 cases among 958,960 controls; the crude relative rate among male immigrants was 0.582.. Comparing immigrants to controls in all age and sex stratified strata, the log rank test *p* < 0.0001 except for females aged > = 75 years at index, where *p* = 0.01. The age-adjusted hazard ratio (HR) for CRC among female immigrants was 0.63 (95% CI 0.59, 0.67) during the first 10 years, and 0.66 (95% CI 0.59, 0.74) thereafter. Among male immigrants the age-adjusted HR = 0.55 (95% CI 0.52, 0.59) during the first 10 years and increased to 0.63 (95% CI 0.57, 0.71) thereafter. The adjusted HR > = 1 only among immigrants born in Europe and Central Asia.

**Conclusions:**

The risk of CRC among immigrants to Ontario relative to controls varies by origin and over time since immigration.

## Background

The incidence of CRC varies between males and females, among individual nations and regions of the world, and is associated with environmental, behavioural and genetic factors [1–3]. Among nations with previously lower incidence and current adoption of Western lifestyles, the incidence is increasing [4–7], although India is a notable example of an exception to this trend [8]. This trend has also been described among immigrants to California from previously lower incidence nations [9].

The incidence of CRC has been high in Canada compared to most other countries. Crude and world age standardized CRC incidence for Canada in 2012 are 68.5 and 35.2 per 100,000 respectively, compared to 50.7 and 27.0 per 100,000 respectively for Poland, 18.6 and 14.2 per 100,000 respectively for China, and 5.1 and 6.1 per 100,000 for India [10]. Lower risk of CRC among immigrants compared to non-immigrant populations with high prevalence of CRC has been described in Canada [11, 12], the USA [13], and the UK [14].

We aimed to compare the risk of CRC among immigrants to Ontario, Canada, to its general population, and to examine if the risk changed over time since arrival, stratified by world region of birth and country of birth of immigrants. Ontario recently established a CRC screening program, ColonCancerCheck (CCC), [15], consisting of biennial guaiac fecal occult blood testing (gFOBT) for persons 50 - 74 without a first degree relative affected by CRC (approximately 89% of the population of Ontario), and screening colonoscopy for those with an affected first degree relative (approximately 11% of the population of Ontario) [16, 17]. CCC sends letters to all 50–74 year old residents of Ontario inviting them to discuss CRC screening with their primary care providers, who are supplied with gFOBT sampling kits to distribute to their eligible patients. It is already known that immigrants to Ontario are less likely to participate in colorectal screening than non-immigrants [18], as is true elsewhere in North America and Europe [19–22]. The goal of this work is to inform CCC of the risk of CRC stratified by origin of immigrants and time since immigration, so that its efforts to improve screening participation among immigrants may be tailored by this information.

## Methods

This work was approved by the Research Ethics Board of Sunnybrook Health Sciences Centre and conducted at the Institute for Clinical Evaluative Sciences (ICES). We used three population-wide databases, in which each observation is identified by an encryption of the unique Ontario Health Insurance Number and are thereby linkable deterministically. The Immigration, Refugees and Citizenship Canada Permanent Resident Database (IRCC) contains the date of arrival in Canada, the intended province of residence in Canada, and the country of birth; permission to access the data was granted by the Government of Canada. The Registered Persons Database (RPDB) contains the age, sex, dates of eligiblity for the universal, single-payer Ontario Health Insurance Plan (OHIP), date of last contact with health care services in Ontario, and status on the date of last contact for each OHIP beneficiary; permission to access the data was granted by the Ministry of Health and Long Term Care of Ontario. The Ontario Cancer Registry (OCR) contains the diagnosis code for invasive cancer (International Classification of Diseases version 10) and its date of diagnosis, for all residents of Ontario diagnosed with a malignancy; permission to access the data was granted by Cancer Care Ontario.

We used an exposure-control matched design. We identified persons from the IRCC with arrival in Ontario, and whose first eligibility for OHIP fell between July 1, 1991 and June 30, 2008 at age 40 years or older, in the Registered Persons Database (RPDB). The first eligibility date was labeled the index date for each immigrant. From the IRCC, we extracted ‘country of birth’ for each immigrant, and categorized ‘country of birth’ into a modified classification of selected world regions (East Asia and Pacific; Europe and Central Asia; Latin America and Caribbean, Middle East and North Africa, South Asia, Sub-saharan Africa) [23–25]. We matched 5 controls from the RPDB alive on the corresponding immigrant’s index date and not found in the IRCC database between 1986 to 2010, on year of birth and sex.

We identified CRC (diagnosis codes C180, C182 - C189, C19, C20) from the OCR among immigrants and controls between the index date and December 31, 2014. All immigrants and controls were followed to date of last contact, date of CRC diagnosis, or December 31, 2014, whichever came first.

We examined the distributions of various characteristics between immigrants and controls in the matched cohort. All analyses were stratified by sex. The crude rates of CRC (per 100,000 person-years) among immigrants and controls, along with the corresponding crude relative rate of CRC, were calculated.

To examine the association between immigration status and the hazard of CRC, the outcome was defined as the time to diagnosis of CRC (from the index date). Individuals were censored at the time of study end or death, whichever occurred first. Kaplan Meier methods were used to graphically examine the risk of CRC over time among immigrants compared to controls, and log-log plots were used to assess if the hazard functions were proportional. Multivariable extended Cox regression models were then implemented, controlling for baseline characteristics [26]. Note that since we performed exposure-control matching, as opposed to case-control matching, the matched characteristics are permitted in the multivariable model. To account for within-cluster correlation that may arise due to the matched design, a robust sandwich variance estimation approach was used. As it was possible for the association between immigration status and hazard of CRC to change over time, we included an interaction between immigration status (immigrant or non-immigrant) and time, where time was categorized into two intervals using the point of 10 years after index [27]. Analyses were conducted with SAS version 9.3 (SAS Institute, Inc., Cary, NC). All statistical tests were two sided, and *P* values less than .05 were considered statistically significant.

## Results

We identified 209,843 female immigrants with 2,538,966 person-years of follow-up to death, diagnosis of CRC, or December 31, 2014, whichever came first. The index date for 84.4% of female immigrants fell between July 1, 1991 and December 31, 2005 (i.e. between 9 and 23 years prior to the last available date for records of CRC diagnosis). We matched them to 1,049,215 controls with 13,917,936 person-years of follow-up. We identified 191,792 male immigrants (for whom the index date fell between July 1, 1991 and December 31, 2005 among 84.7%) with 2,228,448 person-years of follow-up and matched them to 958,960 controls with 12,160,208 person-years of follow-up (Table 1). Mean and median duration follow-up of followup are modestly longer for controls compared to immigrants; this is due to higher emigration from Ontario among immigrants compared to controls, rather than higher mortality. The crude relative death rate among immigrants compared to controls is 0.555 per 100,000 person-years, and is lower among all strata of age at index and among all world regions and countries of birth of immigrants (data not shown).

**Table 1:** Description of immigrants and controls

Females	Immigrants	Controls
Overall count		
Overall count	209,843	1,049,215
Year of index date		
1991 - 1995	59,210 (28.2%)	290,050 (28.2%)
1996 - 2000	53,063 (25.3%)	265,315 (25.3%)
2001 - 2005	64,922 (30.9%)	324,610 (30.9%)
2006 - 2008	32,648 (15.6%)	163,240 (15.6%)
Person years by year of index date		
1991 - 1995	940,939 person-years	5,292,802 person-years
1996 - 2000	725,818 person-years	3,915,579 person-years
2001 - 2005	646,525 person-years	3496,374 person-years
2006 - 2008	225,685 person-years	1,213,181 person-years
Followup time by year of index date		
1991 - 1995		
Mean (SD)	15.89 years (7.59)	17.88 years (6.03)
Median (IQR)	19.76 years (9.88 - 21.55)	20.28 years (16.26 - 21.78)
1996 - 2000		
Mean (SD)	13.68 years (5.18)	14.76 years (4.12)
Median (IQR)	15.40 years (13.08 - 17.27)	15.76 years (14.31 - 17.44)
2001 - 2005		
Mean (SD)	9.96 years (3.48)	10.77 years (2.62)
Median (IQR)	10.84 (9.24 - 12.43)	11.19 years (9.78 - 12.56)
2006 - 2008		
Mean (SD)	6.91 years (2.09)	7.43 years (1.45)
Median (IQR)	7.44 years (6.71 - 8.27)	7.64 years (7.02 - 8.35)
Age at index date		
40 - 49 years	97,829 (46.6%)	489,110 (46.6%)
50 - 59 years	51,806 (24.7%)	259,489 (24.7%)
60 - 69 years	41,029 (19.6%)	204,985 (19.5%)
70 - 74 years	10,342 (4.9%)	51,447 (4.9%)
> = 75 years	8837 (4.2%)	44,184 (4.2%)
Person years by age at index date		
40 - 49 years	1,235,292 person-years	6,646,937 person-years
50 - 59 years	664,305 person-years	3,624,482 person-years
60 - 69 years	470,317 person-years	2,700,938 person-years
70 - 74 years	100,008 person-years	578,455 person-years
> = 75 years	69,043.82 person-years	367,123.65 person-years
Followup time by age at index date		
40 - 49 years		
Mean (SD)	12.63 years (5.78)	13.59 years (5.25)
Median (IQR)	12.34 years (8.44 - 17.27)	13.17 years (9.37 - 17.83)
50 - 59 years		
Mean (SD)	12.82 years (6.24)	13.97 years (5.56)
Median (IQR)	12.63 years (8.25 - 18.24)	13.56 years (9.39 - 18.82)
60 - 69 years		
Mean (SD)	11.46 years (6.46)	13.18 years (5.70)
Median (IQR)	11.42 years (6.87 - 16.66)	12.90 years (8.72 - 17.86)
70 - 74 years		
Mean (SD)	9.67 years (6.16)	11.24 years (5.53)
Median (IQR)	9.32 years (4.57 - 14.15)	10.98 years (7.30 - 15.17)
> = 75 years		
Mean (SD)	7.81 years (5.28)	8.31 years (5.04)
Median (IQR)	7.34 years (3.39 - 11.48)	7.89 years (4.30 - 11.70)
Males	Immigrants	Controls
Overall count	191,792	958,960
Year of index date		
1991 - 1995	48,793 (25.4%)	243,965 (25.4%)
1996 - 2000	51,328 (26.8%)	256,640 (26.8%)
2001 - 2005	62,371 (32.5%)	311,855 (32.5%)
2006 - 2008	29,300 (15.3%)	146,500 (15.3%)
Person years by year of index date		
1991 - 1995	744,331 person-years	4,121,283 person-years
1996 - 2000	685,887 person-years	3,702,915 person-years
2001 - 2005	601,385 person-years	3,273,836 person-years
2006 - 2008	196,843 person-years	1,062,171 person-years
Followup time by year of index date		
1991 - 1995		
Mean (SD)	15.25 years (7.73)	16.89 (6.58)
Median (IQR)	19.45 years (8.28 - 21.30)	19.78 (13.12 - 21.56)
1996 - 2000		
Mean (SD)	13.36 years (5.32)	14.43 (4.34)
Median (IQR)	15.22 years (11.58 - 17.15)	15.56 (14.16 - 17.32)
2001 - 2005		
Mean (SD)	9.64 years (3.69)	10.50 (2.88)
Median (IQR)	10.62 years (9.03 - 12.36)	11.04 (9.50 - 12.50)
2006 - 2008		
Mean (SD)	6.72 years (2.26)	7.25 (1.66)
Median (IQR)	7.37 years (6.58 - 8.24)	7.56 (6.90 - 8.31)
Age at index date		
40 - 49 years	106,112 (55.3%)	530,757 (55.3%)
50 - 59 years	40,062 (20.9%)	200,563 (20.9%)
60 - 69 years	31,575 (16.5%)	157,362 (16.4%)
70 - 74 years	8070 (4.2%)	40,492 (4.2%)
> = 75 years	5973 (3.1%)	29,786 (3.1%)
Person years by age at index date		
40 - 49 years	1,289,734 person-years	7,047,301 person-years
50 - 59 years	485,469 person-years	2,620,618 person-years
60 - 69 years	344,004 person-years	1,886,805 person-years
70 - 74 years	69,618 person-years	390,565 person-years
> = 75 years	39,621 person-years	214,918 person-years
Followup time by age at index date		
40 - 49 years		
Mean (SD)	12.15 years (5.85)	13.28 years (5.23)
Median (IQR)	12.12 years (8.09 - 16.70)	13.09 years (9.31 - 17.36)
50 - 59 years		
Mean (SD)	12.12 years (6.11)	13.07 years (5.57)
Median (IQR)	11.90 years (7.75 - 17.18)	12.61 years (8.69 - 17.70)
60 - 69 years		
Mean (SD)	10.89 years (6.39)	11.99 years (5.77)
Median (IQR)	10.81 years (6.36 - 15.76)	11.65 years (7.81 - 16.43)
70 - 74 years		
Mean (SD)	8.63 years (5.80)	9.65 years (5.29)
Median (IQR)	8.16 years (3.81 - 12.62)	9.11 years (6.09 - 13.08)
> = 75 years		
Mean (SD)	6.63 years (5.03)	7.22 years (4.66)
Median (IQR)	6.18 years (2.18 - 9.90)	6.90 years (3.39 - 10.31)

Among female immigrants, 58.2% had been born in the East Asia and Pacific or the South Asia world regions, and 146,545 / 209,843 (69.8%) had been born in one of 14 among all 211 represented countries of birth, with 27.2% having been born in China / Hong Kong / Taiwan and 20.3% in India. Among male immigrants, 59.3% had been born in the East Asia and Pacific or South Asia world regions, and133,234 / 191,792 (69.5%) had been born in one of 14 among all 213 represented countries of birth, with 27.0% having been born in China / Hong Kong / Taiwan and 22.9% in India (Table 2). Among females, 1877 cases of CRC were observed among immigrants and 16,517 among controls; the crude rate of CRC among female immigrants was 0.203 per 100,000 person-years, compared to 0.325 per 100,000 person-years among female controls, increased by age at index date, and varied by world region and by country of birth Overall, the crude relative rate of CRC among female immigrants was 0.623, ranging from 0.233 among those born in South Asia to 1.027 among those born in Europe and Central Asia. The crude relative rate exceeded 1.000 for those born in Russia and Ukraine. Among males, 1956 cases of CRC were observed among immigrants and 18,329 among controls; the crude rate among immigrants was 0.24 per 100,000 person-years, compared to 0.413 among controls, increased by age at index date, and varied by world region and country of birth. Compared to male controls, the overall crude relative event rate among male immigrants was 0.582. Among world regions, the crude relative rates of CRC for immigrants ranged from 0.218 for those born in South Asia to 1.024 for Europe and Central Asia. The crude relative rate of CRC exceeded 1.000 for those born in Poland, Russia, and Ukraine (Table 2).

**Table 2:** Crude rates of CRC among immigrants and controls and crude relative rate among immigrants

Females	Immigrants	CRC among immigrants	Controls	CRC among controls	Crude rate among immigrants^a^	Crude rate among controls^a^	Crude relative rate among immigrants^a^
Overall	209,843	1877	1,049,215	16,517	0.203	0.325	0.623
Age at index date							
40 - 49 years	97,829 (46.6%)	442 (23.5%)	489,110 (46.6%)	3180 (19.3%)	0.098	0.131	0.748
50 - 59 years	51,806 (24.7%)	428 (22.8%)	259,489 (24.7%)	4299 (26.0%)	0.177	0.325	0.543
60 - 69 years	41,029 (19.6%)	606 (32.3%)	204,985 (19.5%)	6027 (36.5%)	0.353	0.611	0.577
70 - 74 years	10,342 (4.9%)	200 (10.7%)	51,447 (4.9%)	1720 (10.4%)	0.548	0.815	0.673
> = 75 years	8837 (4.2%)	201 (10.7%)	44,184 (4.2%)	1291 (7.8%)	0.798	0.963	0.828
Selected world regions of birth	205,469	1854	1,027,345	16,217			
East Asia and Pacific	67,385 (32.8%)	748 (40.4%)	336,925 (32.8%)	5771 (35.6%)	0.253	0.345	0.731
Europe and Central Asia	38,315 (18.7%)	599 (32.3%)	191,575 (18.7%)	3107 (19.2%)	0.340	0.331	1.027
Latin America and the Caribbean	23,210 (11.3%)	191 (10.3%)	116,050 (11.3%)	1789 (11.0%)	0.176	0.310	0.569
Middle East and North Africa	16,222 (7.9%)	106 (5.7%)	81,110 (7.9%)	1114 (6.9%)	0.155	0.298	0.519
South Asia	52,103 (25.4%)	162 (8.7%)	260,515 (25.4%)	3764 (23.2%)	0.072	0.308	0.233
Sub-saharan Africa	8234 (4.0%)	48 (0.3%)	41,170 (4.0%)	672 (4.1%)	0.134	0.344	0.389
Selected nations of birth	146,545	1230	732,725	11,565			
India	29,744 (20.3%)	73 (5.9%)	148,720 (20.3%)	2173 (18.8%)	0.059	0.315	0.187
Pakistan	7907 (5.4%)	18 (1.5%)	39,535 (5.4%)	435 (3.8%)	0.056	0.247	0.226
Sri Lanka	10,256 (7.0%)	48 (3.9%)	51,280 (7.0%)	893 (7.7%)	0.093	0.343	0.270
China / Hong Kong / Taiwan	39,886 (27.2%)	535 (43.5%)	199,430 (27.2%)	3679 (31.8%)	0.308	0.364	0.845
Philippines	16,461 (11.2%)	125 (7.6%)	82,305 (11.2%)	1232 (10.7%)	0.171	0.316	0.542
Korea	4194 (2.9%)	25 (2.0%)	20,970 (2.9%)	239 (2.1%)	0.145	0.249	0.585
Iran	6754 (4.6%)	40 (3.3%)	33,770 (4.6%)	443 (3.8%)	0.139	0.287	0.484
Poland	5373 (3.7%)	83 (6.8%)	26,865 (3.7%)	544 (4.7%)	0.287	0.351	0.818
Guyana	5072 (3.5%)	30 (2.4%)	25,360 (3.5%)	447 (3.9%)	0.116	0.333	0.349
Jamaica	5012 (3.4%)	65 (5.3%)	25,060 (3.4%)	427 (3.7%)	0.260	0.332	0.783
United States of America	3906 (2.7%)	18 (1.5%)	19,530 (2.7%)	258 (2.2%)	0.127	0.279	0.454
Russia	4686 (3.2%)	70 (5.7%)	23,430 (3.2%)	288 (2.5%)	0.352	0.283	1.244
United Kingdom	3734 (2.6%)	43 (3.5%)	18,670 (2.6%)	299 (2.6%)	0.292	0.356	0.819
Ukraine	3560 (2.4%)	57 (4.6%)	17,800 (2.4%)	208 (1.8%)	0.367	0.264	1.389
Males							
Overall	191,792	1956	958,960	18,329	0.240	0.413	0.582
Age at index date							
40 - 49 years	106,112 (55.3%)	580 (29.7%)	530,757 (55.3%)	4758 (26.0%)	0.123	0.185	0.666
50 - 59 years	40,062 (20.9%)	476 (24.3%)	200,563 (20.9%)	4671 (25.5%)	0.269	0.488	0.550
60 - 69 years	31,575 (16.5%)	610 (31.2%)	157,362 (16.4%)	6237 (34.0%)	0.486	0.906	0.536
70 - 74 years	8070 (4.2%)	168 (8.6%)	40,492 (4.2%)	1676 (9.1%)	0.661	1.176	0.562
> = 75 years	5973 (3.1%)	122 (6.2%)	29,786 (3.1%)	987 (5.4%)	0.844	1.258	0.670
Selected world regions of birth	187,835	1923	939,175	17,999			
East Asia and Pacific	56,546 (30.1%)	787 (40.9%)	282,730 (30.1%)	6209 (34.5%)	0.330	0.464	0.711
Europe and Central Asia	32,489 (17.3%)	570 (29.6%)	162,445 (17.3%)	2968 (16.5%)	0.386	0.377	1.024
Latin America and the Caribbean	17,474 (9.3%)	136 (7.1%)	87,370 (9.3%)	1591 (8.8%)	0.174	0.385	0.452
Middle East and North Africa	18,355 (9.8%)	164 (8.5%)	91,775 (9.8%)	1589 (8.8%)	0.221	0.389	0.570
South Asia	54,756 (29.2%)	205 (10.7%)	273,780 (29.2%)	5025 (27.9%)	0.090	0.413	0.218
Sub-saharan Africa	8215 (4.4%)	61 (3.2%)	41,075 (4.4%)	617 (3.4%)	0.180	0.329	0.545
Selected nations of birth	133,234	1341	666,170	13,209			
India	30,557 (22.9%)	98 (7.3%)	152,785 (22.9%)	3028 (22.9%)	0.079	0.450	0.177
Pakistan	10,875 (8.2%)	34 (2.5%)	54,375 (8.2%)	697 (5.3%)	0.078	0.291	0.268
Sri Lanka	8005 (6.0%)	58 (4.3%)	40,025 (6.0%)	930 (7.0%)	0.150	0.489	0.307
China / Hong Kong / Taiwan	35,957 (27.0%)	562 (41.9%)	179,785 (27.0%)	4246 (32.1%)	0.376	0.495	0.759
Philippines	11,588 (8.7%)	115 (8.6%)	57,940 (8.7%)	1152 (8.7%)	0.229	0.433	0.528
Korea	4257 (3.2%)	41 (3.1%)	21,285 (3.2%)	252 (1.9%)	0.234	0.255	0.920
Iran	7381 (5.5%)	86 (6.4%)	36,905 (5.5%)	636 (4.8%)	0.279	0.385	0.724
Poland	3343 (2.5%)	70 (5.2%)	16,715 (2.5%)	373 (2.8%)	0.383	0.377	1.017
Guyana	3601 (2.7%)	31 (2.3%)	18,005 (2.7%)	427 (3.2%)	0.176	0.471	0.374
Jamaica	3496 (2.6%)	31 (2.3%)	17,480 (2.6%)	335 (2.5%)	0.184	0.394	0.466
United States of America	3506 (2.6%)	28 (2.1%)	17,530 (2.6%)	289 (2.2%)	0.230	0.372	0.618
Russia	3662 (2.8%)	71 (5.3%)	18,310 (2.8%)	256 (1.9%)	0.463	0.316	1.464
United Kingdom	4142 (3.1%)	62 (4.6%)	20,710 (3.1%)	370 (2.8%)	0.373	0.405	0.921
Ukraine	2864 (2.1%)	54 (4.0%)	14,320 (2.1%)	218 (1.7%)	0.437	0.344	1.269

^a^per 100,000 person-years

By the Kaplan-Meier method, the risk of colorectal cancer diagnosis over time was consistently higher among female controls compared to immigrants in all age strata (log rank test *p* < 0.0001 except for females > = 75 years of age, log rank test *p* = 0.01), and among male controls compared to immigrants (log rank test *p* < 0.0001 in all age strata). Examination of sex and age stratified log-log plots of time to colorectal cancer demonstrated that the hazards of CRC were proportional in all sex and age strata. (not shown).

The age-adjusted hazard ratio (HR) for CRC among female immigrants was 0.63 (95% CI 0.59, 0.67) during the first 10 years after arrival, and 0.66 (95% CI 0.59, 0.74) thereafter. Among male immigrants the age-adjusted HR = 0.55 (95% CI 0.52, 0.59) during the first 10 years, and 0.63 (95% CI 0.57, 0.71) thereafter.

**Fig. 1:**
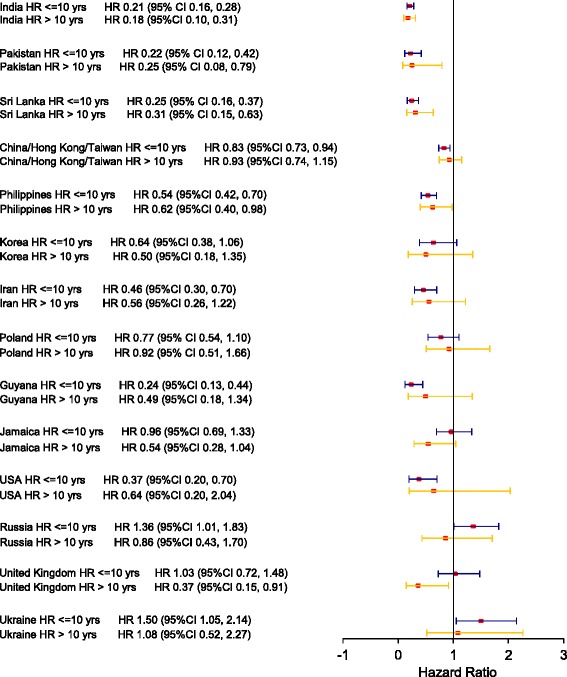
Forest plot for Adjusted Hazard Ratio for CRC among females by birth country

**Fig. 2:**
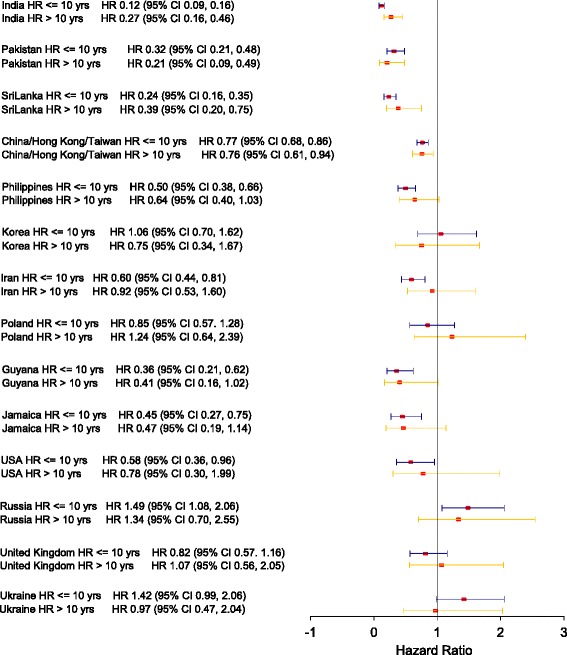
Forest plot for Adjusted Hazard Ratio for CRC among males by birth country

The age-adjusted HRs vary among the world regions, and in general are significantly less than 1.00 except among those born in Europe and Central Asia. The point estimates of the age-adjusted HRs > 10 years after the index date are increased compared to <= 10 years for some regions and the respective 95% confidence intervals include unity. Figures 1 and 2 display forest plots of the stratified age-adjusted HRs for CRC for the 14 countries of birth with the largest number of immigrants, for females and males respectively. There are greater differences in age-adjusted HRs among these 14 countries of birth compared to those among the seven world regions of birth in Table 3.

**Table 3:** Adjusted cox models of CRC risk stratified by sex

Variable	Females			Males		
	Univariate HR (95% CI)	Multivariate HR (95% CI)	Multivariate HR (95% CI) with interaction	Univariate HR (95% CI)	Multivariate HR (95% CI)	Multivariate HR (95% CI) with interaction
Overall						
Immigrant	0.63 (0.60, 0.66)	0.64 (0.61, 0.67)		0.59 (0.56, 0.62)	0.59 (0.56, 0.62)	
Nonimmigrant controls	Reference	Reference		Reference	Reference	
Age at index						
40 - 49	Reference	Reference	Reference	Reference	Reference	Reference
50 - 59	2.35 (2.25, 2.45)	2.35 (2.25, 2.45)	2.35 (2.25, 2.45)	2.59 (2.50, 2.70)	2.60 (2.50, 2.70)	2.60 (2.50, 2.70)
60 - 69	4.54 (4.36, 4.73)	4.53 (4.35, 4.72)	4.53 (4.35, 4.72)	4.94 (4.77, 5.12)	4.94 (4.77, 5.12)	4.94 (4.77, 5.12)
70 - 74	6.54 (6.19, 6.91)	6.52 (6.17, 6.88)	6.52 (6.17, 6.88)	6.99 (6.62, 7.37)	6.98 (6.62, 7.37)	6.98 (6.62, 7.37)
> = 75	8.63 (8.11, 9.17)	8.63 (8.12, 9,17)	8.63 (8.12, 9.17)	8.46 (7.93, 9.03)	8.47 (7.94, 9.04)	8.47 (7.94, 9.04)
Immigrant < = 10 years since index date	0.62 (0.58, 0.66)		0.63 (0.59, 0.67)	0.55 (0.52, 0.59)		0.55 (0.52, 0.59)
Controls <= 10 years since index date	Reference		Reference	Reference		Reference
Immigrant > 10 years since index date	0.65 (0.58, 0.73)		0.66 (0.59, 0.74)	0.64 (0.57, 0.72)		0.63 (0.57, 0.71)
Controls <= 10 years since index date	Reference		Reference	Reference		Reference
Age adjusted models for world region of birth						
East Asia and Pacific						
Immigrant < = 10 years since index date	0.70 (0.63, 0.78)		0.72 (0.65, 0.80)	0.70 (0.64, 0.78)		0.72 (0.65, 0.79)
Controls <= 10 years since index date	Reference		Reference	Reference		Reference
Immigrant > 10 years since index date	0.79 (0.66, 0.95)		0.80 (0.67, 0.96)	0.74 (0.62, 0.89)		0.73 (0.61, 0.87)
Controls <= 10 years since index date	Reference		Reference	Reference		Reference
Europe and Central Asia						
Immigrant< = 10 years since index date	1.10 (0.98, 1.23)		1.09 (0.97, 1.22)	1.02 (0.90, 1.15)		1.01 (0.90, 1.14)
Controls <= 10 years since index date	Reference		Reference	Reference		Reference
Immigrant > 10 years since index date	0.96 (0.77, 1.18)		0.96 (0.78, 1.19)	1.05 (0.85, 1.30)		1.04 (0.84, 1.29)
Controls <= 10 years since index date	Reference		Reference	Reference		Reference
Latin America and the Caribbean						
Immigrant < = 10 years since index date	0.58 (0.47, 0.71)		0.59 (0.48, 0.72)	0.45 (0.35, 0.57)		0.46 (0.36, 0.58)
Controls <= 10 years since index date	Reference		Reference	Reference		Reference
Immigrant > 10 years since index date	0.56 (0.39, 0.81)		0.58 (0.41, 0.84)	0.46 (0.30, 0.71)		0.46 (0.30, 0.71)
Controls <= 10 years since index date	Reference		Reference	Reference		Reference
Middle East and North Africa						
Immigrant< = 10 years since index date	0.52 (0.40, 0.67)		0.52 (0.40, 0.68)	0.49 (0.39, 0.61)		0.48 (0.38, 0.60)
Controls <= 10 years since index date	Reference		Reference	Reference		Reference
Immigrant > 10 years since index date	0.54 (0.33, 0.87)		0.54 (0.34, 0.88)	0.72 (0.49, 1.07)		0.70 (0.47, 1.03)
Controls <= 10 years since index date	Reference		Reference	Reference		Reference
South Asia						
Immigrant < = 10 years since index date	0.23 (0.19, 0.28)		0.24 (0.19, 0.29)	0.17 (0.14, 0.21)		0.18 (0.14, 0.22)
Controls <= 10 years since index date	Reference		Reference	Reference		Reference
Immigrant > 10 years since index date	0.24 (0.17, 0.35)		0.25 (0.17, 0.37)	0.28 (0.20, 0.40)		0.28 (0.20, 0.40)
Controls <= 10 years since index date	Reference		Reference	Reference		Reference
Sub-saharan Africa						
Immigrant < = 10 years since index date	0.27 (0.17, 0.43)		0.27 (0.17, 0.43)	0.39 (0.26, 0.59)		0.38 (0.25, 0.57)
Controls <= 10 years since index date	Reference		Reference	Reference		Reference
Immigrant > 10 years since index date	0.56 (0.26, 1.18)		0.56 (0.26, 1.18)	0.78 (0.40, 1.53)		0.75 (0.38, 1.48)
Controls <= 10 years since index date	Reference		Reference	Reference		Reference

## Discussion

The risk of CRC among immigrants who arrived in Ontario at the age of 40 years or older, between 1991 and 2008, is lower overall compared to age-matched controls for more than 10 years after immigration. Their risk varies among world regions of birth, and among the 14 countries in which the majority of immigrants were born. All arrived in Ontario prior to the inception of the CCC screening program and many of those diagnosed with CRC received the diagnosis prior to its inception. It is already known that immigrants to Ontario are less likely to participate in CRC screening [18]. In its future efforts to improve participation in CRC screening by immigrants, CCC should include tailored approaches to the sizable numbers of immigrants from countries of birth for which the age-adjusted risks of CRC are close to, or equivalent to, those of the non-immigrant controls.

This is an important study comprising a large population of 401,635 immigrants from 213 countries. The available data elements are appropriate for a time-to-event analytic approach, that is more powerful and illuminating than crude rates and incidence ratios, and it was possible to examine the potential interaction between status as an immigrant and time since immigration. Prior studies of the risk of CRC among immigrants have substantial weaknesses compared to this work. A national study among immigrants to Canada from 1980 to 1990, using probabilistic rather than deterministic linkage, with followup for CRC among other cancer types to 1998, had fewer immigrants despite being nation-wide, 90.8% of whom were <= 44 years of age at the time of immigration [11]. The young age distribution is reflected in the low number of cases of CRC observed among immigrants (*n* = 71 compared to *n* = 3833); the low number of cases prevented the use of time to event analytic methods and stratification by country of birth. Other Canadian studies of small numbers of immigrants were unable to use to time to event methods and did not examine the worldwide distribution of origin of immigrants [12, 28]. Studies from the US [13] and the UK [14] examined relative rates between immigrants from a limited number of origins.

A limitation of the study is the inability to match immigrants to controls on comorbidity or past medical history on the matching date. Although immigrants and controls hypothetically could be linked to census-level ecologic measures of socio-economic status by postal code on the matching date, we doubt that those measures at the time of the arrival of immigrants in Ontario would be valid descriptors of socio-economic influences on the health of the immigrants up to the time of their arrival. A further limitation is the lack of data about the risks of CRC beyond two decades since arrival, which might plausbility increase as observed among certain ethnic groups in California [9]. We are unable to comment on the association of colorectal screening with immigration status, or the association between colorectal screening among immigrants and their risk of colorectal cancer in this study, because the population-based CRC screening programme in Ontario was introduced near the end of the observation period.

The variability of the risk of CRC among immigrants to Ontario relative to controls, by region or country of origin, has implications for colorectal screening in Ontario. Efforts to improve screening participation among immigrants may be tailored by this information. Furthermore, the risk of CRC should be monitored during the period beyond two decades since arrival.

## Conclusions

The risk of CRC among immigrants to Ontario varies by length of time residing in Ontario and birthplace, and, with the exception of those born in the Europe andCentral Asia region, and in the individual countries of Russia and Ukraine, is lower than that for non-immigrants for up to two decades after arrival.

## Abbreviations

CCC: Colon Cancer Check
CI: Confidence interval
CRC: Colorectal cancer
HR: Hazard ratio
ICES: Institute for Clinical Evaluative Sciences
IRCC: Immigration, Refugees and Citizenship Canada
OCR: Ontario Cancer Registry
OHIP: Ontario Health Insurance Plan
